# Supplementary material for: A dynamic model to sustain the spark: How do network coordinators in Dutch healthcare networks maintain network commitment?
Source: PLoS One. 2025 Jul 22;20(7):e0326915. doi: 10.1371/journal.pone.0326915 (PMC12282924; doi:10.1371/journal.pone.0326915)
Supplement: S2 Appendix — (PDF) [file pone.0326915.s003.pdf]

Topic Guide	
**1. Introduction**	- Can you briefly introduce yourself and your professional background?<br>- What is your age and relevant work experience?
**2. Network Experience**	- What kind of healthcare improvement network are you part of?<br>- What is the size and composition of these networks?<br>- Which organizations or partners were/are involved?<br>- What were the goals of the networks and how did you aim to achieve them?
**3. Process and Phases of Change**	- What phases or steps would you distinguish in the process toward achieving healthcare improvement?<br>- On which levels (individual, organizational, network) do these play out?
**4. Achieving Change as a Network**	- To what extent did the networks succeed in bringing about change?<br>- Did you apply a specific method or approach?
**5. Barriers and Challenges**	- What were the main difficulties or barriers in achieving change as a network?<br>- How did you address these challenges in practice?
**6. Enablers of Change**	- What supported successful change?<br>- What characteristics or capacities were helpful—at the individual, organizational, or network level?<br>- What kind of resources were necessary?
**7. Contextual Factors**	- What contextual factors affected the network's ability to implement change?<br>- How did you deal with these contextual influences?
**8. Closing Questions**	- Do you have any suggestions for improving this topic guide?<br>- Do you know other people we could interview, such as members of improvement networks?
