# Supplementary material for: A dynamic model to sustain the spark: How do network coordinators in Dutch healthcare networks maintain network commitment?
Source: PLoS One. 2025 Jul 22;20(7):e0326915. doi: 10.1371/journal.pone.0326915 (PMC12282924; doi:10.1371/journal.pone.0326915)
Supplement: S3 Appendix — (PDF) [file pone.0326915.s004.pdf]

Network Characteristics

Context of the Networks

Network Goals

- Make basic data available in blinded
- Make eHealth or questionnaires available in blinded
- Exchange data between organizations, national level
- Regional change projects
- Partnerships: Elderly care / GP, hospitals, insurers
- Value based care

Network Size

Network Origin

Participation Requirements

Stages of Change

- Executive Phase
- Development Phase
- Pilot Phase
- Implementation Phase
- Consolidation Phase
- Structuring and Adjusting

Factors Promoting and Hindering Change Efforts

Common Goals and Approach

- Flexible strategy documents
- Common annual plan, approach alignment

Problem Solving

- Iterative approach of identifying and solving problems

- Discussing solutions within the network participants

Adaptability

- Adjusting to context and circumstances

Lead Team

Learning from Other Organizations

Factors Influencing Commitment, Support, and Motivation

Common Goal

- Inspiring goals and joint strategy

Involvement of Management/Board

Motivation

- Intrinsic motivation
- Sharing results to maintain motivation
- Small, cyclical implementation of changes

Multidisciplinary Collaboration and Involvement

Role Division and Dynamics in the Network

- Assigning tasks to the right people

Multidisciplinary Collaboration

Joint Action for Change

Involving the Target Group in the Network

Practical Conditions

- Transferability
- Financial Resources and Personnel Capacity
- Government and Policy Support
- Obligation and Accessibility
- Lack of Consolidation

Results

- Improvement of care related outcomes
- Support from the target group
- Scaling of initiatives

Challenges Perceived by Network Coordinators

- Alignment between institutions
- Decision on expanding the network and its effect on dynamics
- Determining how to sustain the change
- Large-scale changes affect all participating organizations
- Goals are not a linear cause-effect process, difficult to evaluate
- Dealing with declining energy and motivation in the network
- Integrating changes into hospitals
- Making sure change becomes part of daily work
- Deciding in which care sectors to scale up
- Introducing innovation to the public and ensuring usage
- Not all best practices can be copied due to context differences
- Projects and organizations are different, policies must adapt
- Dilemma of success for the organization versus the network
- Keeping people committed
- How to create movement and change with so many partners
- How to deal with faster and slower moving parties
- How to develop a common strategy
- How to keep the network unique and valuable over time
- Formalizing the network or not
- Balancing different interests of network members
- Evaluating improvements and scientifically proving results
