# Supplementary material for: A dynamic model to sustain the spark: How do network coordinators in Dutch healthcare networks maintain network commitment?
Source: PLoS One. 2025 Jul 22;20(7):e0326915. doi: 10.1371/journal.pone.0326915 (PMC12282924; doi:10.1371/journal.pone.0326915)
Supplement: Attachment — Submitted filename: Response letter 2.docx [file pone.0326915.s006.docx]

Dear editor,

We have now attached the original ethics approval document, which was approved on 20^th^ of April 2022, along with the research protocol (in English) for ethics approval for the study.

We would like to clarify that **no** extension to the ethics approval was made for the reported study. The current paper fits within the scope of the original ethics approval, and all the methods used, including the semi-structured interviews, were part of the initial study design and ethical approval.

To provide further clarification, we would like to highlight how the submitted paper fits within the broader study design outlined in our ethics proposal. As detailed in the ethical approval, the study encompasses multiple objectives and methods, including action research aimed at improving multidisciplinary discussions, anticoagulation care, and care for vulnerable elderly patients. A key focus of this research is on the long-term effectiveness of these initiatives, particularly within the context of Learning & Working Labs, in networks.

The current paper is part of this larger research objective, specifically focusing on the long-term effectiveness of the networks established through these Labs. In line with the approved study design, we employed semi-structured interviews as one of our primary methods, which is explicitly covered in the ethics proposal.

We have now ensured that the correct documents and relevant explanations are included. We hope this additional context clarifies the alignment between our paper and the ethical approval.

Please let us know if there is anything further required.

Best regards,

Manon Roest, M.D.

On behalf of the co-authors
