# Supplementary material for: A dynamic model to sustain the spark: How do network coordinators in Dutch healthcare networks maintain network commitment?
Source: PLoS One. 2025 Jul 22;20(7):e0326915. doi: 10.1371/journal.pone.0326915 (PMC12282924; doi:10.1371/journal.pone.0326915)
Supplement: Attachment — Submitted filename: Response letter to the reviewers.docx [file pone.0326915.s007.docx]

Dear editor, and both reviewers,

We sincerely appreciate the thorough and insightful comments, which have greatly contributed to enhancing the quality of our paper. Below, we provide a detailed response to each of the reviewers’ comments. Please note that some comments overlap, and in such cases, we have provided a more concise response, referring to previous answers. Should any further clarification be required, we would be happy to provide additional information.

**Reviewer #1**

1. Comment: Thank you for the opportunity to review this paper on commitment for healthcare workers. The paper has an interesting aim: how do healthcare networks with long-term quality improvement goals manage to keep participants committed?

- We want to thank you for taking the time to evaluate our paper and for recognizing the interesting aim of our study. We also like to thank you for your thorough and insightful comments, which have greatly contributed to enhancing the quality of our paper. Over the past weeks, we have carefully refined, restructured and clarified various sections to strengthen our arguments. Several comments from you and the other reviewers touched on the issue of alignment and overlap. Therefore, we chose to synthesize the feedback, resulting in substantial changes throughout the manuscript. We focused on enhancing the clarity, coherence and alignment of our arguments. One key improvement from our point of view was the integration of a theoretical framework in the discussion section. This helped to structure and visualize our findings. We believe that it improved the overall readability and quality of the manuscript and hope the revisions align with your expectations.

2. Comment: In method section, please define criteria for inclusion of healthcare networks.

- Thank you for pointing this out to us that we need to include specific information on this. We have 1) elaborated on the selection process and the types of networks included in our study. To provide greater clarity, we have 2) separated the inclusion criteria from the data collection section, ensuring a clearer distinction between both processes. Additionally, we have 3) expanded on the types of networks included in our study by detailing their healthcare improvement objectives and 4) clarified in text that our interviewees were network representatives responsible for managing the networks, rather than frontline healthcare professionals. Additionally, we have 5) added this (meaning 1-4) information to the characteristics table (table 1.) as well. By doing so we have provided a more detailed explanation of the networks included in our study, while ensuring the anonymity of our study objects. The latter was somewhat challenging as, within the Dutch context, there are few national or regional networks with similar objectives, making it important to balance transparency with confidentiality.
- Adjustment to the manuscript: see line 112 – 177.

3. Comment: Please give a more in dept description of the included healthcare networks. Was it nurses or doctors, nurses or others? Or a mix.

- Thank you for this suggestion, as it makes clear which types of networks we included. We have now clarified the types of professionals involved in these networks and have added this to both the characteristics table 1 and the main text, while maintaining participant anonymity.
- Adjustment to manuscript: Line 135-136, 147-149. (Table 1)

4. Comment: The method section is not comprehensive please expand.

- Thank you for your feedback, in response to this comment and other comments regarding the method section we have made several revisions. We have expanded the inclusion criteria, made a clearer structure by separating inclusion criteria and data collection. We have provided more details of the included networks and explain that we interviewed network representatives, mainly network leaders or directors, rather than frontline healthcare professionals. We have expanded our description of the initiative Time to Connect, outlining its role in enhancing patient safety through networks, in the most concise way. Additionally, we have elaborated on the coding steps and how we identified the themes.
- Adjustment: line 112 - 174

5. Comment: Please include more information about the pilot.

- Thank you for this suggestion, we agree that providing additional context about the Time to Connect program is important for understanding both the broader focus of the initiative and the specific scope of our study. Since this program resulted in the selection of specific types of networks included in our research, healthcare networks that improve care, we have added a more detailed yet concise description of the program.
- Adjustment: line 120 - 126

6. Comment: Definition on network and how you identify that about active healthcare need to be expaned. Perhaps a figure to visualize this?

- Thank you for this important observation. We agree that the concept of a network should be introduced earlier. We adhere to *Provan and Kenis’(2008)* definition, which states that a network consists of at least three or more organizations that collaborate toward a common goal. Furthermore, we have restructured the introduction to first establish the importance of networks in addressing healthcare challenges (line 35-43), followed by a concise elaboration of networks in the Dutch healthcare context, including a case of a network that aims to improve care quality together (line 44-58). This leads into our argument of sustaining networks over time and the role of network management (60-88), particularly in managing network commitment (90-102). We believe that this revised structure ensures a logical flow and more clarity on the concepts.

7. Comment: What is an extended network please define.

- We interpret the question as referring to our use of the term “extended period” (*previous* line 269) in relation to the duration of the network operations. In our study, we have used this term to describe the long-term aspect of the networks included, meaning, networks that operate over an extended period rather than short-term collaborations. This long-term aspect results from their healthcare improvement objectives, which are broad and complex, requiring multistakeholder involvement and collaboration over time. Achieving change/improvement takes time, not only implementing improvement but also institutionalizing them within the healthcare system. Thus, when we referred to extended networks, we meant that these networks should function over a longer period to ensure that improvements become embedded in practice. We have removed this sentence, altered the section and now clarified the definition of networks and their healthcare improvement objectives.
- Adjustment: Line 44-58.

8. Comment: How was the themes defined?

- We have provided a clearer explanation of how the themes were identified and refined. The thematic analysis followed the six steps of *Braun and Clarke (2006)*. Initially, the transcripts were openly coded which resulted in four preliminary themes. Using axial coding these themes were refined, through an iterative process and relationships between codes were discussed. Eventually we restructured the content to distinguish between the reasons for declining network commitment and the approaches network managers employed to manage commitment.
- Adjustment in text: line 162 – 172 (data analysis)

9. Comment: How can healthcare workers use this paper – consider to include that in the discussion. And what kind of healthcare worker?

- Thank you for this helpful suggestion. We have clarified the practical relevance of our study in both the introduction and discussion section. In the introduction we now highlight that a deeper understanding of how networks function and evolve is essential for network managers, this includes healthcare professionals that are given such roles. Additionally, in the discussion, we included a visual framework (adaptation of Ring and Van de Ven)which helps to illustrate the complexity and interdependency of managing network commitment over time. This framework is not only our theoretical contribution, but it can also offer practical guidance for those involved in the coordination, facilitation, or participation in healthcare networks.

Please read the new Discussion as we revised and restructured this section.

**Reviewer #2**

1. Comment: The topic is highly interesting and the qualitative research is an appropriate design to explore in depth the inner working mechanisms of how a health network by be sustained from the perspective of actors, however, in general, the paper lack in depth description and conceptual clarification to make sense of the different social mechanisms identified (trust, reciprocity, stakeholders' involvement, alignment of goals and so on). Please see some references that may or may not be helpful in addressing some conceptual clarity on the complex intertwined relationships underlying the long-term commitment of actors in healthcare networks!

- We sincerely thank the reviewer for their time and detailed feedback. We appreciate the recognition of the relevance of our topic and the suitability of our qualitative design for exploring how network coordinators managed to sustain network commitment in healthcare improvement networks. While our primary focus has been on capturing the practical experiences of network coordinators, we agree that conceptual grounding of key mechanisms, such as trust, reciprocity, stakeholder involvement, and goal alignment, enhances the richness of our paper. The reviewer’s comments thus prompted us to restructure the manuscript and substantially revise the discussion, where we now offer a more conceptual clarification and visualize the interdependencies between managerial actions and social mechanisms, moving beyond merely stating their existence. Because of the detailed feedback and suggested literature we had the opportunity to restructure our paper and particularly enhanced the discussion where we made more conceptual clarification and explained and visualized the interdependencies of managerial actions and social mechanisms by adapting Ring and Van de Ven, more than just stating there is interdependency between factors. We have also used the refences provided by the reviewer.
- Adjustment: please read the full Discussion section, as this is a completely new section.

2. Comment: (abstract) Background: Insufficient specification of the rationale of the study, which seems like a single research question done in non-specified context which is necessary to make sense of the research question. The research questions need to stem from the problem specification and a research gap. No mention of the methods, data collection tools, sites, and qualitative data analysis used? Results What do interorganizational healthcare networks with long-term improvement goals stem from? This was not specified and clarified in the background. The results section needs to answer the question about the drivers of the maintenance of long-term commitment. The results seem vague and do not relate to the question raised: What are the drivers of the maintenance of long-term commitment? Discussion : The discussion asserts that the study addresses why and how networks wane. This does not align with the research question raised! And this was not addressed, at least in the results section of the manuscript. The recommendations, as well as the policy and practical implications, are rather vague. What does a nuanced approach mean?

- We thank the reviewer for the detailed and constructive comments on the abstract. We have substantially revised the abstract to ensure it accurately reflects the updated and restructured manuscript. In the background, we have now clearly stated the rationale, within the Dutch context and a better connection with the research question. The methods now include key methodological details. The results have been restructured and revised to directly respond to the research question. In the discussion we now introduce the adjusted framework and the importance of timing managerial actions. We have also removed vague statements, such as “nuanced approach”.
- Adjustments: please read the new abstract as this is a completely new section.

3. Comment: Recommendation : Please make sure the research question raised aligns with the results presented and the practical and policy implications. Stating that it is interdependent is not a real insight, as you mention that healthcare networks are complex and address complex wicked challenges. The definition of complexity includes non-linearity, interdependency, and emergence, among other characteristics.’

- Thank you for this valuable recommendation, we have realigned the research question and the results section. Specifically, we revised it in the manuscript to the following: (lines 104-110):

*“This study aims to empirically explore if, and why the network commitment of participants declined and how network commitment was managed by network coordinators in Dutch healthcare improvement networks. By exploring the factors influencing the development of network commitment and the approaches coordinators used to maintain engagement, this research will provide insight into the management of network commitment within the Dutch healthcare context. “*

Furthermore, the result section has been restructured to reflect the two core components of our research question: 1) The reasons for declining network commitment of network participants and 2) the approaches used by network coordinators to manage network commitment in their network.

- Regarding the point on interdependency, we fully agree that simply stating its presence in healthcare networks offers limited value, especially given the nature of complex systems. Rather than treating “interdependency” as a standalone insight, we have made an effort to illustrate when specific managerial actions – such as setting preconditions upfront- influences the decline in network commitment, which is itself shaped by social mechanisms as trust. We have integrated our exploratory findings into the developmental framework of Ring and Van de Ven, which allowed us to visualize the interdependencies and the temporal dimension of managing network commitment of participants across different stages of collaboration. Please read the discussion and see figure 1 for these adjustments.

4. Comment: No pagination of the manuscript.

- We understand that no pagination may make it more challenging to review the manuscript. We appreciate the reviewer bringing this to our attention. The manuscript now contains pagination.

5. Comment: Page 9, line 33 : The framing of sentences needs some English editing.

- Thank you for pointing this out. In revising the manuscript, we have paid close attention to the overall language clarity and sentence structure. Therefore, we made numerous changes in the whole manuscript and asked an English editor to carefully check the paper.
- Adjustment: See the track changes version.

6. Comment: "Given the complexity of the problems?" What problems?

- We agree that the original phrasing could have been clearer in linking the term ‘complex problems” to specific challenges. We have restructured the introduction to first outline specific challenges faced by healthcare systems – such as the rising demand for services while containing costs and ensuring a suitable work environment for professionals. Calling these ‘wicked problems’ was based on previous work by *Provan and Lemaire* *(2012)* and *O’Toole (1997).* We have however, removed “wicked” and focused on describing the nature of the challenges more clearly.
- See for example adjustments: line 35-40

7. Comment: The competitiveness of goals is not a problem; it is the very nature of healthcare organizations, which have always had paradoxical goals (see Parsons' social systems and Cameron and Quinn’s competitive culture framework).

- Our intention was not so much to frame goal competitiveness as a problem in itself, but rather to acknowledge that goals need to be aligned in order to be an effective network , that practical challenges emerge when goals are aligned across diverse organizations and how this affects network commitment and goal attainment, at the start of the network but also later on. We have elaborated more on inter-organizational networks and their stability and sustainability.
- Adjustment: line 60 – 88

8. Comment: No rationale underlying the study of network commitment. The objective in the abstract states the exploration of the drivers of the maintenance of long-term commitment. The first paragraph states the relationship between network commitment and care quality, which is a different objective.

- Thank you for your thorough reading and remark on misalignment in writing. We have realigned the research question, results and conclusion throughout the manuscript to ensure consistency. We have also revised the introduction to articulate the relevancy of studying network commitment as part of managing networks in this particular context. We believe this provides a stronger rationale and thank you for your help.
- Please read the introduction as we completely revised this section.

9. Comment: The introduction of the Dutch network is abrupt. A careful introduction to the Dutch health system is needed before addressing healthcare network challenges and then the objective of the study.

- We understand your need to understand the Dutch healthcare system more in depth. Therefore, the specific Dutch context, is introduced in the revised manuscript in the Introduction, where we outline the collaborative initiatives to foster collaborative learning and improvement through networks that are present in the Dutch healthcare system. Also, we introduced the reason for undertaking this study as our contribution to the Time to Connect program. We have also included a practical example (see line 46-49) of a regional networking the Netherlands which requires active engagement of multiple stakeholders.
- Adjustments: line 43-52

10. Comment: Paragraph, line 45: The relationship between commitment and trust is bidirectional, not linear. So the authors might use a less prescriptive affirmation: "Clearly, network commitment is essential to network processes and goal attainment."

- You raise a valid observation; our original phrasing was too affirmative and may have implied a one-directional relationship. We have removed this statement from the manuscript.

11. Comment: Paragraph, lines 55-56: The research gap is not well defined, and the reference used to justify the research gap is a cross-sectional study on the perceived importance of healthcare networks among healthcare alliances in the US. More systematic reviews and scoping reviews are needed to justify this specific research gap, as well as contextual evidence from Europe and the Dutch context in particular.

- Thank you for this comment. We have now revised the introduction section (and abstract part) with a better focus on the necessity of network commitment (line 60-88) and the challenges of managing networks including network commitment (line 90-102).

12. Comment: What motivated the research? What practical challenges does the Dutch health(care) system face?

Examples can be: van der Weert, G., Burzynska, K., & Knoben, J. (2022). An integrative perspective on interorganizational multilevel healthcare networks: a systematic literature review. BMC Health Services Research, 22(1), 923.
BELRHITI, Z., BIGDELI, M., LAKHAL, A., DIB, K., ZBIRI, S., & BELABBES, S. (2024). Unravelling collaborative governance dynamics within healthcare networks: a scoping review. Health Policy Planning (in press).
CARMONE, A. E., KALARIS, K., LEYDON, N., SIRIVANSANTI, N., SMITH, J. M., STOREY, A., & MALATA, A. (2020). Developing a common understanding of networks of care through a scoping study. Health Systems & Reform, 6, e1810921.
DE POURCQ, K., DE REGGE, M., VAN DEN HEEDE, K., VAN DE VOORDE, C., PAUL, G., & EECKLOO, K. (2019). The role of governance in different types of interhospital collaborations: a systematic review. Health Policy, 123, 472-479.

- As stated earlier, we agree that a stronger contextualization of the Dutch healthcare system contributes to the relevance of our work, which we have now incorporated in the introduction. We also appreciate the suggested literature of the reviewer. And used van *Van der Weert, G., Burzynska, K., & Knoben, J. (2022)*, *Belrhiti et al., (2024)* and *Carmone et al., (2020)* as reference to contextualize our explorative findings in the discussion section. For the introduction we have used additional references including *Van de Bovenkamp et al. (2017)* on governance and regulation in an institutionally layered healthcare system, and *Van der Woerd et al. (2024)* on regional network-building for complex care delivery.

13. Comment: The authors use the term healthcare network with long-term quality improvement goals—are there any other forms of healthcare networks? Who has developed this typology? Is this stated in policy documents? In that case, please ensure the Dutch healthcare system's context is well clarified to allow the reader to make sense of what is presented.

- Thank you for this thoughtful and important comment. We acknowledge that there are indeed different types of networks described, such as managed networks, cooperative networks, developmental networks or agency networks (*Randall, 2013; Malby&Wallace, 2016; Provan&Milward 1995; Mandell&Keast 2007*). Moreover, literature on purpose-oriented networks (*Provan&Kenis 2008, Peeters 2022*) can be seen as closely aligned with the types of networks studied in our paper, particularly given their effort to reach a shared goal. However, we did not want to elaborate on existing typologies in the main text, since this would shift the emphasis away from the practice-oriented perspective of our paper. Our aim was not to develop or discuss the typology of healthcare networks nor to contribute to governance theory. Rather we focus on empirically exploring how network coordinators manage participant commitment within networks that operate in the Dutch healthcare system with quality improvement goals. Therefore, we have taken explain in the introduction and methods the term healthcare improvement networks by referring to inter-organizational collaborations aimed at quality improvement which takes time and asks for long-term commitment.

14. Comment: COREQ I have no access to Appendix 1. Appendix need to be numerated accordingly 1, 2 and so on not appendix 1 and then appendix A there need also a pagination.

- We have now ensured that all appendices are consistently numbered using a numerical format, rather than a mix of numerical and alphabetical labels. We have also added pagination on both manuscript and appendices to improve clarity and navigation.

15. Comment: Material and methods. The rationale behind the framing of the research question still needs to be well formulated and sufficiently enriched with contextual data from the Netherlands' healthcare system and how this fits into the policies, organizational, and quality-of-care challenges.

- As noted in earlier responses, we have now provided a more clear rationale for our research questions in both the Introduction section and methods section. Additionally, we have outlined the challenges faced worldwide which call for network collaboration, including the Netherlands.

16. Comment: Network commitment needs to be defined—what does this mean? Is it about long-term or short-term commitment? Then, of whom? Partners? Healthcare providers? Managers? Health professionals?

- In the revised manuscript we have now expanded the Introduction section to provide a clearer explanation of the concept of network commitment. We now define network commitment of encompassing both active engagement and psychological attachment to the network *(Clarke, 2006)*. We explain that these components are essential in preventing passive involvement, fostering decision-making and sustaining effective collaboration *(Clarke , 2006; Clarke, 2005; Morgan &Hunt, 1994)*. While we do not explicitly distinguish between short-term and long-term commitment in theoretical terms, the context of our study – networks with healthcare improvement objectives – focuses on the need for sustained commitment over time, as we explain the complex challenges faced as well. We have stressed this context in the introduction.
- See lines 60-110

Regarding *who* is expected to be committed: Our study focuses on how network managers overseeing their networks and respond to challenges – particularly the challenge of declining commitment among network participants over time. In the method section we have made more explicit that we interviewed representatives of networks, these networks had different characteristics including different participants such as healthcare professionals or managers.

- Adjustments: lines 146-148

17. Comment: Only in the Material and Methods section do the authors state that this research is a commissioned project. This needs to be stated in the Introduction section after the contextual specification and a rich description of the healthcare system where this study is undertaken.

- We have now described the national Time to Connect (Tijd voor Verbinding) program, which was initiated to enhance patient safety through collaborative learning in healthcare networks in the Methods section. We also explain the rational (origin and relevance) of the study as it is connected with the Dutch healthcare context.
- Adjustments: line 47 – 50 and 114-116.

18. Comment: "Data collection" rather than "data gathering"!

- We have adjusted the heading and now use data collection.

19. Comment: What does long-term objectives mean for the selection of cases?

- In the method section, we now clarify that we used purposive sampling to identify networks in the Netherlands that focused on complex healthcare improvement goals involving multiple organizations. These goals extended beyond short-term gains or shared saving objectives. The focus was on enhancing patient safety, and advancing multidisciplinary collaboration by setting up networks for knowledge sharing and learning. We first mapped existing networks and then approached them to request participation. Due to the limited number of such networks in the Netherlands, we cannot provide further specification regarding the networks, their participants, or their objectives, as doing so would compromise participant anonymity.
- Adjustments: 128 - 135

20. Comment: What is the unit of analysis?

- The unit of analysis in this study are the approaches and practices of network coordinators in managing healthcare improvement networks. The data were drawn from interviews with representative of the network in managerial or coordinating roles and the analysis centered on the challenges they faced, the strategic actions and responses they described. We explained this better in the methods section.
- Please read the new method section as this is a completely new section.

21. Comment: What is the case (see *Yin, 2018*, definition of a case)?

- Following the definition of a case as a “contemporary phenomenon within its real-life context” (*Yin, 2018*), we define each case in our study as an instance of a networks coordinator’s management practice within a healthcare improvement network with long-term objectives. The phenomenon we explore is the networks management approaches, specifically those to maintaining participant commitment while the network is operational. The context is the Dutch healthcare system. In this multiple case study approach we explored various strategies and their commonalities.

22. Comment: What does active healthcare networks mean? How was this assessed? Is there any database of key performance indicators underlying the purposive sampling?

- In our study, the term “active healthcare network” refers to networks that were currently operational at the time of data collection. These networks were identified based on public information, professional networks, and the employees of the program of Time to Connect who are embedded in the Dutch healthcare system. We did not rely on a centralized of key performance indicators for sampling. We explained better in the Methods section our sampling strategy.
- Adjustment: line 134-144

23. Comment: In purposive sampling, what are the selection criteria? What is the principle used in qualitative sampling (deviant cases? Positive cases? Negative cases?) (see Patton, 2001).

- The selection criteria included variation in network size, scope (regional vs. national), governance structures, funding arrangements and participation requirements. We aimed for diversity which allowed us to identify challenges and strategies across different settings. In line with Patton’s typology of qualitative sampling strategies, our approach can best be described as a maximum variation sampling, which is one of the purposive sampling methods described*. (Patton, 2002).* We did not specifically focus on deviant, positive or negative cases, as our goal was to capture a broad spectrum of experiences and to synthesize them.
- Adjustment: lines 134 - 135

24. Comment: I have no access to Appendix A! Why is the number of the appendix "1" and then "A"? The uploaded manuscript does not include appendices! I am not able to check the interview guide!

- I am sorry to hear that you were not able to assess all the appendices. We have now ensured consistent numbering of all appendices and double checked if the interview guide is in the system.

25. What is obligatory involvement? Were the healthcare networks mandatory or voluntary? These aspects need to be well defined in the Introduction!

- We have clarified in the method section and in the network characteristics table that, while networks were generally voluntary collaborations, some involved a degree of mandatory involvement due to (regional) policy objectives.

26. Comment: Line 121 : The verbatim rather states the need to align motivation with why—the underlying motives for engagement—which was not expressed in the title of this subsection!

- We understand the reviewer’s interpretation of the verbatim quote as pointing towards a deeper exploration of underlying motives for engagement, which is a different study focus. We have chosen to remain with our research questions, which centers on how network coordinators managed participant commitment in healthcare improvement networks. In our study we define network commitment as encompassing both active engagement and psychological attachment to the network *(Clarke 2006)*. From this perspective, participant’s initial motivation to engage is considered an integral part of understanding network commitment. Therefore, we embedded the quote more into the results section.

27. Comment: Line 158: The phenomenon of resistance to change is not sufficiently expressed in operational terms. It is rather described theoretically. Qualitative studies are context-rich, so what are the manifestations of this resistance to change?

- In our revised manuscript, we have restructured the results section into two clear subsection: one focusing on the reasons for declining commitment, and another on the approaches used by the network managers to manage participant commitment. For example, we now describe, how resistance became apparent through hesitancy to implement new practices at the organizational level.
- Adjustments: line 237 - 242

28. Comment: Quote P6a is not really related to resistance to change (the subsection under which it is mentioned). Yet, it rather addresses the strategies to increase the long-term commitment of network partners!

- We agree that in the earlier version, the placement of quote P6a was not clearly aligned with the surrounding text. With the restructured result section this quote is now a better fit under the specific approaches used to maintain network commitment. This illustrates that getting to know one another on a network level improves reliability and responsiveness, and how this supports trust. Moreover, it emphasizes that action (“actually achieving things”) grow out of personal contact.
- Adjustments: see 285-292.

29. Comment: Once again, the categories and themes presented do not align with the research question: drivers for the maintenance of long-term commitment of network partners. Yet, in section line 179 (reciprocity and trust), it is rather about the motivation to join the network!

- We have restructured the result section. Regarding the reciprocity and trust: this is a case-based example illustrating when a lack of reciprocity can arise, for instance when participants have joined the network solely to acquire insights. This lack of reciprocity arises however in a later stage of the network. We hope that the revised manuscript is now clearer.
- Adjustments: 232-236.

30. Comment: There should be a framework used to align the phases of network formation and maintenance to organize the themes into a clear and fluent logical account and narrative.

- We sincerely thank the reviewer for this suggestion. This comment guided us back to our synthesis and ultimately led to the visualization of our explorative findings within a framework in the discussion. We chose to incorporate the framework by Ring and van de Ven, which outlines three cyclical stages of interorganizational cooperation – negotiation, commitment and execution. The framework’s emphasis on the balance between formal and informal processes made it particularly well-suited for visualizing both managerial actions and social mechanisms. In addition, the cyclical nature of the framework aligned well with the ongoing need for network coordination, especially in the context of maintaining participant commitment over time. We mapped the reasons for declining commitment (Rx) and the corresponding approaches (Ax) onto this model. This mapping visualizes the timing and interdependency involved in network management, providing both a more coherent narrative and a practical tool for understanding of how network commitment of participants can be managed in this context.
- Adjustment: line 337 – 391

31. Comment: Yet, the presentation of themes still needs to be reorganized to tell a story rather than a succession of initial coding results

- We have restructured the results and discussion to ensure a more coherent presentation, moving beyond a sequence of coding outcomes. The added framework enabled us to discuss our explorative findings in a more systematic way. To connect the framework in the Discussion section we have included a summary table that organizes the identified reasons for declining commitment and the corresponding approaches.

32. Comment: Line 197 : Participants’ or stakeholders’ engagement as a strategy for long-term engagement is already stated in the previous paragraph. If this is important, reorganize under a subsection (see Emerson’s collaborative governance framework).

- We have reorganized the Result section to improve clarity and flow.

33. Comment: Discussion: The study results did not actually answer the research question: How and why are healthcare networks sustained? Or how is long-term commitment ensured?

- The substantially revised manuscript contains a refined research question and the results and discussion have been realigned accordingly. We have integrated our findings into a framework and included a visualization that links the identified reasons and managerial approaches. This now offers a clear and structured answer to the research question.
- Adjustments: Please read the new Discussion as this is a completely revised section.

34. Comment: Identifying the reasons/contextual conditions reducing long-term commitment is not the same as the reasons underlying the maintenance of healthcare networks!

- Thank you for the comment. We have revised the manuscript so there is no misunderstanding about the scope of the study. To clarify our focus on the practical insight: our aim was to explore how network coordinators managed network commitment in healthcare improvement networks.

35. Comment: What is meant by a nuanced approach?

- Thank you for pointing this out. By “a nuanced approach” in the previous version, we referred to the balancing act network coordinators face in managing healthcare networks, such as finding the right balance between hierarchy and having no-control. We have now clarified this phrasing in the revised discussion.

36. Comment: The authors need to shed light on the interorganizational and intraorganizational contexts that have not been sufficiently described to allow adequate sense-making of the assumed hypotheses of why healthcare networks fail to maintain long-term commitment.

- We have extensively revised the Discussion section to better contextualize our exploratory findings and to clearly position our theoretical contribution. To support this, we have incorporated relevant literature, including the suggested work of *van der Weert, G., Burzynska, K., & Knoben, J. (2022)*, *Belrhiti et al., (2024)*, *Carmone et al., (2020)* as well as *Tunisini and Marchiori (2020)*’s work on why networks fail. These additions contribute to a richer conceptual and contextual foundation. Regarding the inter-organizational and intra-organizational contexts, we acknowledge that commitment is not solely shaped at one level but also influenced by intraorganizational factors. For example, resistance to change can arise within a participating organization, even when initial alignment at the network level exists. We reflect on this in the limitation section, highlighting the need for future research to explore the interplay between intra- and interorganizational processes in healthcare networks.
- Adjustment: see Limitations section

37. The interdependency paragraph needs to use some frameworks to make sense of the collaborative governance complex social dynamics.

- n our revised manuscript, we have addressed this by embedding our findings within the developmental processes framework of Ring and van de Ven. This allowed us to conceptualize and visualize the complex dynamics of collaborative governance, managerial actions and social mechanisms. Also we showed explicit how we address several research gaps identified in recent systematic review papers.

38. Reflectivity of the researchers needs to be included in the reporting, following the COREQ guidelines!

- We have now explained the steps taken in the data collection and analysis phase of this study, showing the reflectivity moments were we discussed up to consensus our findings.
